# Real-time Myocardial Oximetry—Preclinical Monitoring Device Validation in a Porcine Beating-Heart Cardiopulmonary Bypass Model

**DOI:** 10.1016/j.atssr.2026.01.015

**Published:** 2026-02-09

**Authors:** Eetu Haavisto, Henri Haapanen, Liisa Petäjä, Peter Raivio, Esko Kankuri, Mikko Jormalainen, Kalle Kotilahti, Tatu Juvonen, Tommi Pätilä

**Affiliations:** 1 Faculty of Medicine, Department of Pharmacology, University of Helsinki, Helsinki, Finland; 2 Pediatric Cardiac Surgery, Children’s Hospital, Helsinki University Hospital, and University of Helsinki, Helsinki, Finland; 3 Department of Anesthesiology, Heart and Lung Center, University of Helsinki and Helsinki University Hospital, Helsinki, Finland; 4 Department of Cardiac Surgery, Heart and Lung Center, University of Helsinki and Helsinki University Hospital, Helsinki, Finland; 5 Department of Neuroscience and Biomedical Engineering, Aalto University, School of Science, Espoo, Finland; 6 SpectroCor Ltd, Helsinki, Finland

## Abstract

**Purpose:**

Continuous, real-time monitoring of myocardial oxygenation during cardiac surgery holds potential for enhancing patient safety and improving outcomes. This study evaluated the precision of the near-infrared spectroscopy-based SpectroCor Ltd monitoring device, which measures myocardial hemoglobin/myoglobin (Hb/Mb) oxygen saturation and cytochrome c oxidase (CcO) oxidation ratio.

**Description:**

Yorkshire pigs, under general anesthesia, were subjected to cardiopulmonary bypass to generate graded myocardial desaturation. Graded desaturation at aortic root was induced by mixing oxygenated blood from partly pumping cardiopulmonary bypass with deoxygenated blood passing nonventilated lungs. SpectroCor myocardial oximetry was validated against blood samples drawn simultaneously from the aortic root and coronary sinus during various stable saturation intervals.

**Evaluation:**

With accuracy of 5.3 percentage points, the SpectroCor device’s myocardial Hb/Mb oxygen saturation readings tightly correlated with reference blood samples. Concurrent changes in CcO oxidation ratio closely mirrored Hb/Mb saturation, indicating reliable assessment of mitochondrial function.

**Conclusions:**

The SpectroCor monitoring device provides accurate, real-time measurements of myocardial oxygenation, supporting its potential to help optimize cardioprotective strategies during cardiac surgery.

## Visual Abstract

**Figure:**
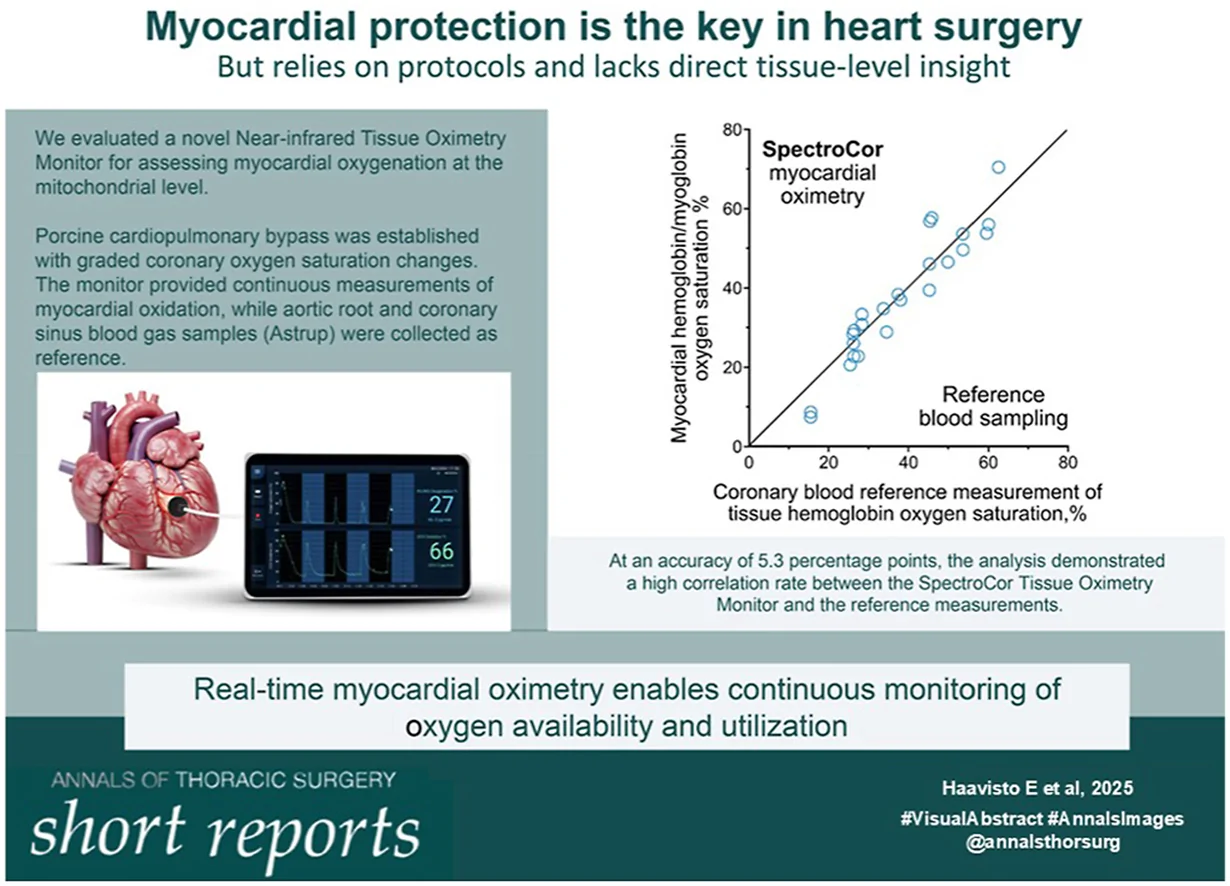

## Technology

Current methods for assessing myocardial health during cardiac surgery are mostly indirect,^1^^,^^2^ and objective direct monitoring of tissue-level oxygenation is unavailable. Tools based on near-infrared spectroscopy are used to noninvasively track cerebral hemoglobin saturations, but do not allow direct evaluation of the myocardial metabolic state and are also limited by wavelength constraints and tissue interference.^3^, ^4^, ^5^

**Figure 1:**
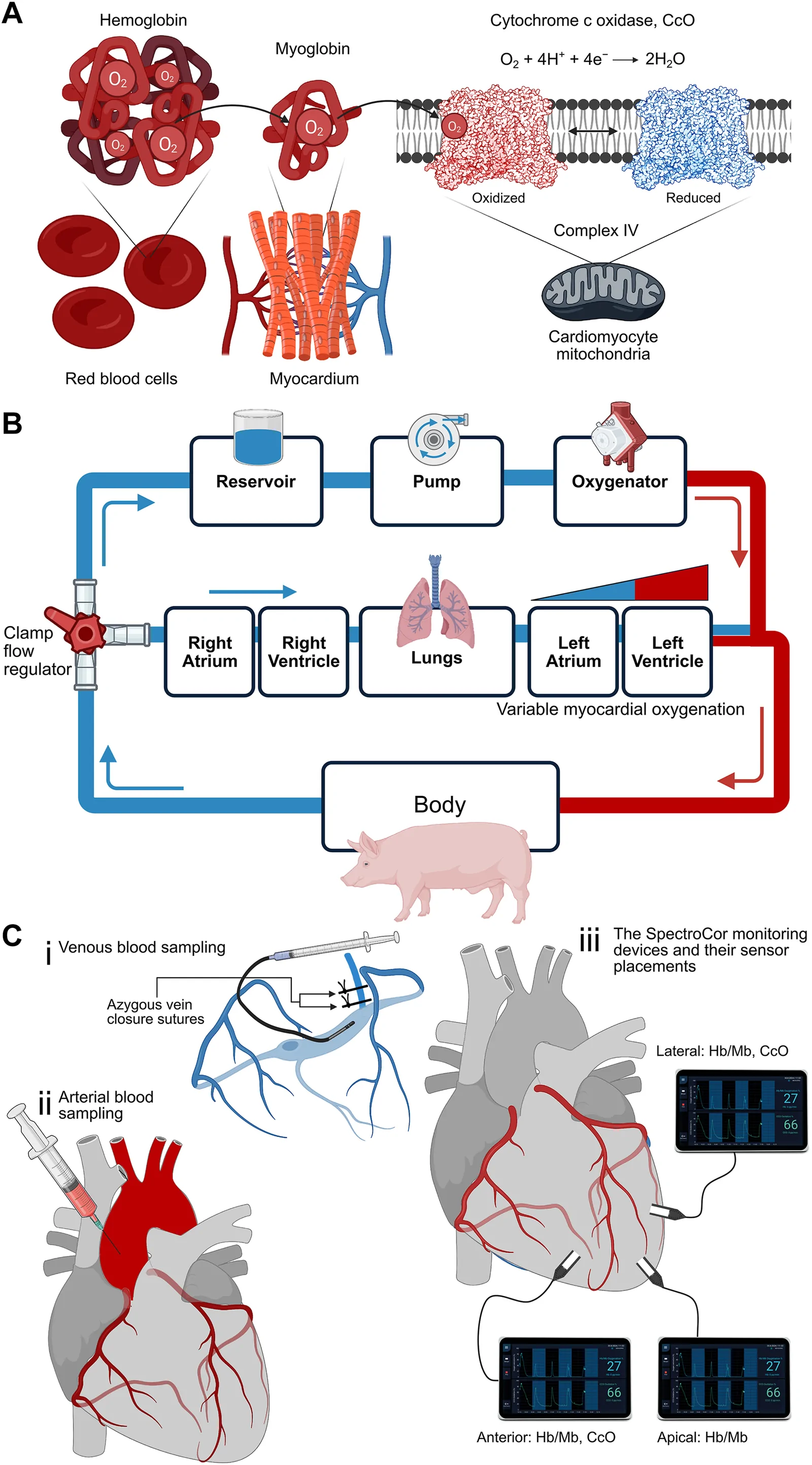
Experimental setup. (A) Oxygen is transported to tissues bound to hemoglobin (Hb). In myocardium, oxygen is transferred to high-oxygen-affinity myoglobin. Within the myocardial cells, cytochrome c oxidase (CcO, complex IV), is the final oxygen reduction site enabling subsequent ATP production and cellular respiration. Structural data: cytochrome c oxidase complex CIV (PDB 2OCC). Myocardium illustration: DataBase Center for Life Science (DBCLS) reproduced under the CC BY 4.0 license from Wikimedia Commons, Curid97833567. (B) Cardiopulmonary bypass setup by cannulation of the right atrium (venous line cannulation) and aorta (arterial line cannulation). A blood reservoir, a pump, and a membrane oxygenator were used to circulate blood, provide gaseous exchange and blood oxygenation. Partial clamping of the venous line restricted blood flow allowing a portion of the venous return to bypass the unventilated lungs and creating a mixture of oxygenated (red) and deoxygenated (blue) blood in the aortic root. (C) Reference blood sampling and placement of SpectroCor sensors. i: To obtain samples reflecting specifically the myocardial oxygen consumption, the left azygos vein was ligated (arrows) to prevent the mixing of lower body venous blood with the coronary venous circulation. ii: Reference blood samples were obtained from the arterial root of the aorta (arterial input) and the coronary sinus (venous output). iii: SpectoCor monitoring device and sensors implanted in the myocardium at anterior wall (Anterior), apex (Apical), and lateral wall (Lateral). (Hb/Mb, hemoglobin/myoglobin; CcO, complex IV.) Images created with BioRender.com.

Heart function relies on oxidative metabolism. Because mitochondria make up about one third of heart volume, to obtain a comprehensive view of both oxygen availability and utilization it is important to measure not just tissue levels of oxygenated hemoglobin (Hb) and myoglobin (Mb), but also the activity of mitochondrial oxidative metabolism. The oxidation state of the terminal mitochondrial site of oxygen reduction, cytochrome c oxidase (CcO), is a critical marker of this process (Figure 1A).^6^

To address the need of myocardial oxygenation monitoring, we developed the SpectroCor monitoring device, a broadband diffuse reflectance spectroscopy device for measuring both myocardial Hb/Mb saturation (S_Hb/Mb_) and CcO redox state (O_CcO_) in real time directly from the myocardium. We already confirmed the device’s high measurement accuracy in a tissue phantom^7^ and aimed in this study to assess and test its ability to detect changes in oxygen saturation and mitochondrial activity under controllably variable ischemic conditions that mimic real life surgical scenarios.

## Technique

All procedures and interventions were carried out according to the Principles of Laboratory Animal Care formatted by the National Society for Medical Research and the Guide for the Care and Use of Laboratory Animals (NIH publication no. 86-23, revised 1985). The study adhered to the ARRIVE 2.0 guidelines and its protocol and procedures were approved by Provincial Government of Southern Finland (ESAVI/17971/2023).

### Anesthesia

Three domestic piglets (57-62 kg) were sedated (intramuscular ketamine 10 mg/kg, midazolam 1.5 mg/kg, medetomidine 0.5 mg/kg) and anesthesia was administered using fentanyl (50 μg/kg), propofol (1.5 mg/kg), and rocuronium (1 mg/kg). Endotracheally intubated animals were ventilated with 50% oxygen, targeting an end-tidal carbon dioxide concentration of 4.5%-5.0%. Continuous infusion of fentanyl (20 μg/[kg×h]), midazolam (45 mg/[kg×h]), rocuronium (0.25 mg/[kg×h]), and ketamine (2 mg/[kg×h]) was used for anesthesia maintenance. During periods of incomplete perfusion, isoflurane inhalation (1.0%-2.5%) anesthesia was administered. Arterial pressure was monitored through an arterial line in the right femoral artery and heart rhythm using a 3-lead electrocardiography system.

### Cardiopulmonary Bypass

Quadrox-i Oxygenator (Getinge AB) was primed with 500 mL of Ringer’s acetate and operated with a Gambro roller pump. After median sternotomy, the animals were heparinized (5000 IU/kg). The right carotid artery was exposed and utilized for arterial line cannulation (22-mm Optisite, Edwards Lifesciences LLC) and the right atrial appendage was used for venous line access (28/36 2-stage venous cannula, Medtronic). Perfusion line temperature was maintained through an oxygenator-connected water coil (Heater, Tekamer). Perfusion was controlled according to cardiac filling (flow rate 3.0-5.0 L/min).

To enable adjustment of myocardial oxygenation, the venous line was partially clamped to reduce drainage, allowing the heart to fill partially and pump some deoxygenated blood through the non-ventilated lungs into the aortic root. Simultaneously, the cardiopulmonary bypass (CPB) system delivered oxygenated blood, creating a controlled mix (Figure 1B).

### Reference Blood Sampling

In porcine anatomy, the left azygos vein drains via the coronary sinus into the right atrium, analogous to a persistent left superior vena cava in humans. Therefore, to prevent systemic venous admixture, the left azygos vein was ligated and the sampling cannula positioned well within the coronary sinus through a purse-string suture, making retrograde right atrial flow highly unlikely in the unloaded heart (Figure 1C). A manual-inflate retroplegia cannula (Terumo Cardiovascular) was inserted into the sinus coronarius for myocardial venous sampling (Figure 1C). A root infusion cannula (Terumo) was inserted into the proximal aortic root for arterial blood sampling (Figure 1C). An ABL90 FLEX PLUS (Radiometer) blood gas analyzer calibrated according to the manufacturer’s instructions was used for reference measurements. Arterial and venous blood samples were collected simultaneously. The ABL90 gives results in 35 seconds, with a 60-second delay between samples. The delay from blood sampling to measurement was kept minimal with the venous sample analyzed first to reduce risk of sample contamination by ambient air oxygen. The reference (S_Reference_) arterial:venous blood volume ratio was set at 20:80 (0.2×S_artery_+0.8×S_vein_).^8^^,^^9^

### Myocardial Spectroscopy

Three SpectroCor monitoring devices (Model 10-00-001, SpectroCor Ltd) and sensor (Model 72-00-007) were used to assess myocardial oxygenation by measuring light absorption across a broad wavelength spectrum (550-900 nm). Three myocardial sensors with light covers were inserted into the left ventricle (Figure 1C). One to the lateral wall (Lateral wall) one to the apex (Apical), and one to the anterior wall (Anterior wall), covered with a light shield to prevent interference from ambient light. The shield was stably affixed to the epicardial surface using *n*-butyl-2-cyanoacrylate tissue adhesive (Histoacryl).^10^ The Anterior and Lateral setups were used for simultaneous S_Hb/Mb_ and O_CcO_ recordings, while the Apical setup recorded S_Hb/Mb_. Each sensor consists of 2 fine measuring needles (∼26 G) securely inserted into the myocardium for optical measurements within the myocardial tissue. The SpectroCor monitoring device’s unique features are shown in Table 1.

**Table 1:** Comparison of SpectroCor Monitoring Device Characteristics With INVOS and ForeSight Near-Infrared Spectroscopy Devices

Characteristic	SpectroCor Monitoring Device (SpectroCor Ltd)	INVOS (Medtronic)	ForeSight (Edwards Lifesciences)
**Wavelength**	Broadband multiple wavelengths within 550-900 nm	Two measuring wavelengths: 730 nm and 810 nm; 4 in total	5 wavelengths: 685, 730, 770, 810, 870 nm (covers 650-900 nm range)
**Measurement location**	Myocardium (in-the-tissue measurement)	Through scalp	Through scalp
**Measurement parameter**	Direct myocardial hemoglobin/myoglobin oxygen saturation and myocardial cytochrome c oxidase for mitochondrial oxygen metabolism	Regional cerebral/somatic oxygen saturation	Regional cerebral/somatic tissue oxygen saturation and relative changes in total tissue hemoglobin
**Measurement area**	Local	Local/regional	Local/regional

### Statistical And Correlation Analyses

The root mean square accuracy (A_rms_) of hemoglobin/myoglobin oxygen saturation was calculated according to the standard EN ISO 80601-2-85:2021 as

$$A_{rms}=\sqrt{\frac{\sum_{i=1}^{m}{\left(S_{Hb/Mb,i}-S_{reference,j\left(i\right)}\right)}^{2}}{m}}$$

where $S_{Hb/Mb,i}$ is the SpectroCor monitor S_Hb/Mb_ measurement corresponding to reference measurement *j*(*i)* (S_reference,j_ = 0.2×S_artery,j_+0.8×S_vein,j_), where S_artery,j_ and S_vein,j_ are ratios of oxyhemoglobin concentration to total hemoglobin concentration reported by the ABL90 FLEX PLUS blood gas analyzer, and *m* is the number of monitor measurement–reference measurement pairs.

Linear correlation between changes in hemoglobin/myoglobin oxygen saturation and changes in O_CcO_ was quantified with a Pearson correlation coefficient derived from the complete dataset without averaging one animal dataset or pooling across animals. Calculation of *P*-values was done under the null hypothesis that the values are uncorrelated.

## Clinical Experience

### Experimental CPB Setup Stability

**Figure 2:**
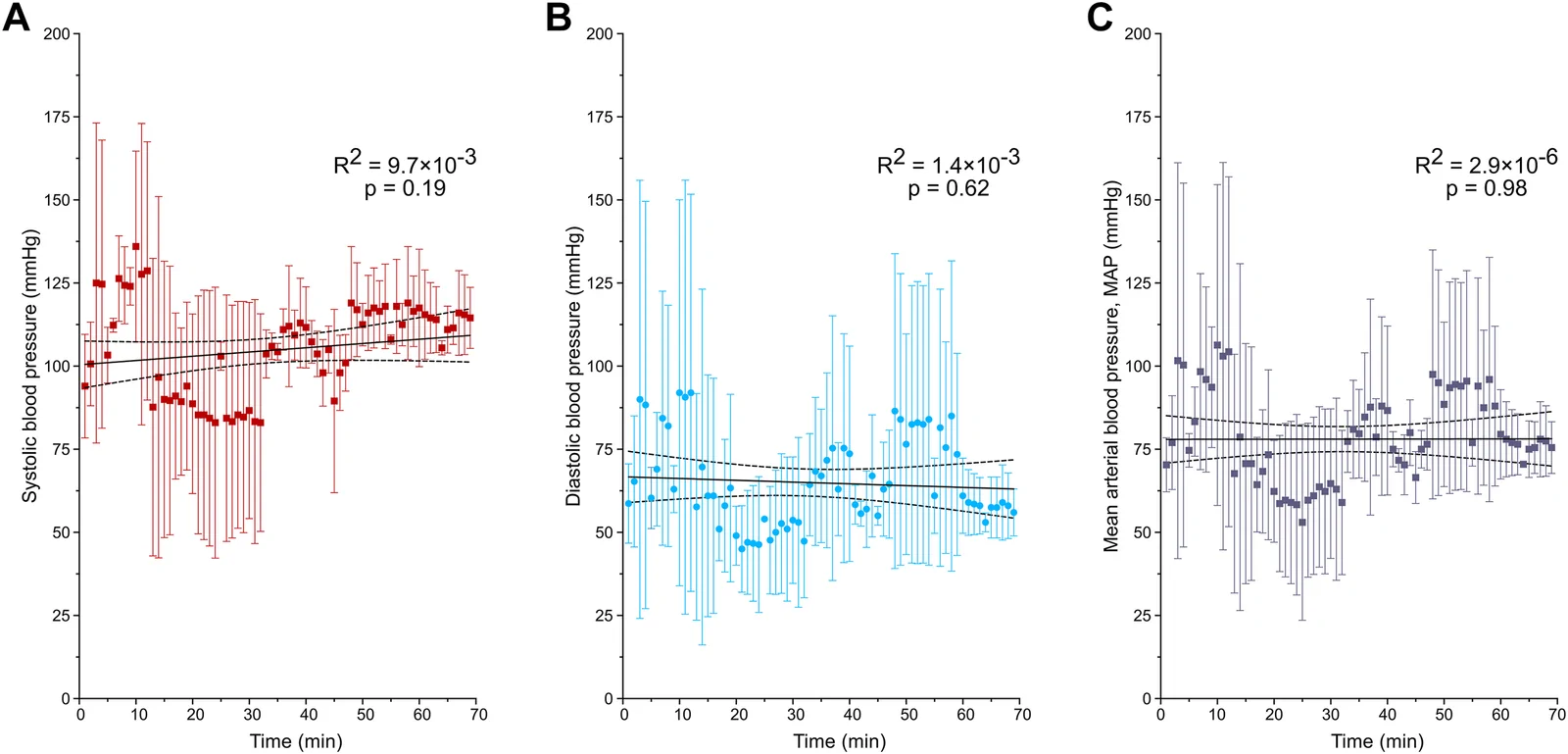
Hemodynamics. (A) Systolic, (B) diastolic, and (C) mean arterial blood pressures (MAP) during the experiments. Data presented as mean ± SD with linear regression (solid lines) and 95% CIs (dashed lines).

Systolic blood pressure (83-136 mm Hg, 105.6 ± 14.1mm Hg, N = 5), diastolic blood pressure (45-92 mm Hg, 65.4 ±13.3 mmHg, N =5), and mean arterial pressure between 53 and 106 mm Hg (78.7 ± 13.4 mm Hg, N =5) (Figure 2) demonstrated the excellent overall stability of the experimental CPB setup.

### Myocardial Oxygenation Monitoring

**Figure 3:**
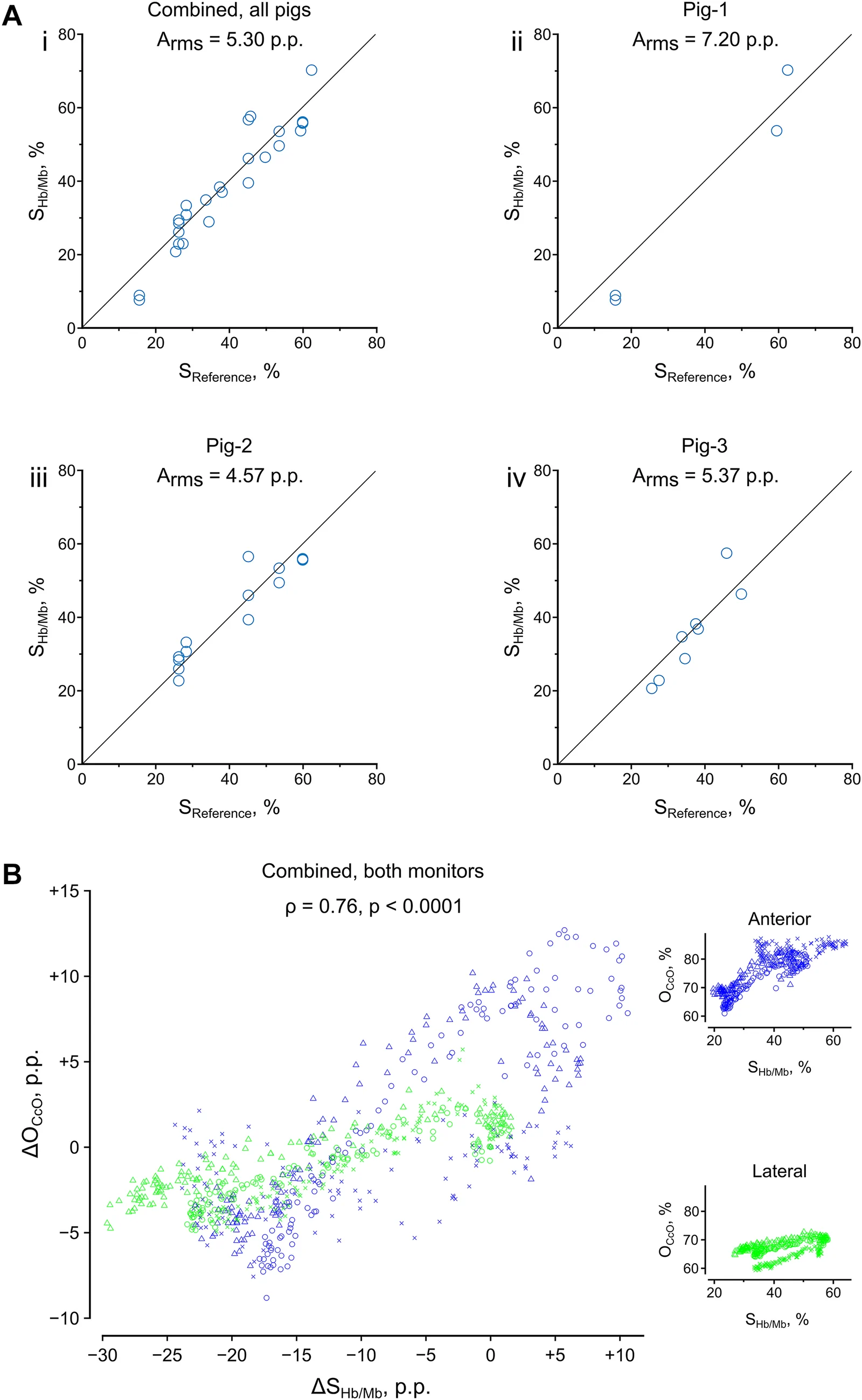
SpectroCor myocardial oximetry. (A) SpectroCor tissue hemoglobin/myoglobin oxygen saturation (S_Hb/Mb_) measurements from all 3 monitors and sensor positions in all three animals (i) and from the differentially placed individual sensors from all three animals (ii–iv) against the reference weighted arterial and venous hemoglobin oxygen saturations from blood gas monitoring data (S_reference_ = 0.2×S_arterial_HbO_2_+0.8×S_venous_HbO_2_) for myocardial tissue oxygenation. Accuracy of the SpectroCor monitoring device was 5.30 percentage points (p.p.). (B) Linear correlation between the changes in cytochrome c oxidase oxidation ratio measurements (ΔO_CcO_) and changes in myocardial hemoglobin/myoglobin oxygen saturation measurements (ΔS_Hb/Mb_) in a single animal. Large panel: Interrelationship (ρ=0.76) between the measurements. Data combined from measurements from two monitors and their respective sensors: Anterior and Lateral. Small panels: Data from sensors (Anterior and Lateral) and CcO oxidation ratio measurements against monitor-measured myocardial hemoglobin/myoglobin oxygen saturation (S_Hb/Mb_). Each repetition is represented by a different symbol (cross, circle, triangle). For clarity, every fifth data point is plotted.

At reference blood sampling, S_Hb/Mb_ and O_CcO_ values from 3 independent myocardial sensors were recorded. Figure 3A shows the comparison and correlations between reference measurements fitted arterial and venous saturation mix (S_Reference_ = 0.2×S_artery_+0.8×S_vein_, ranging from 16% to 63%) and the monitor’s S_Hb/Mb_ (tissue hemoglobin/myoglobin oxygen saturation, ranging from 7% to 70%). At an accuracy of 5.3 percentage points the analysis demonstrated a high correlation rate between the SpectroCor and the reference measurements. The SpectroCor device’s measurements of O_CcO_ and S_Hb/Mb_ correlated well (ρ = 0.76) demonstrating the device’s capability to simultaneously measure oxygen availability and mitochondrial utilization in the beating heart (Figure 3B).

### SpectroCor Sensor Data Repeatability and Real-Time Continuous Monitoring

**Figure 4:**
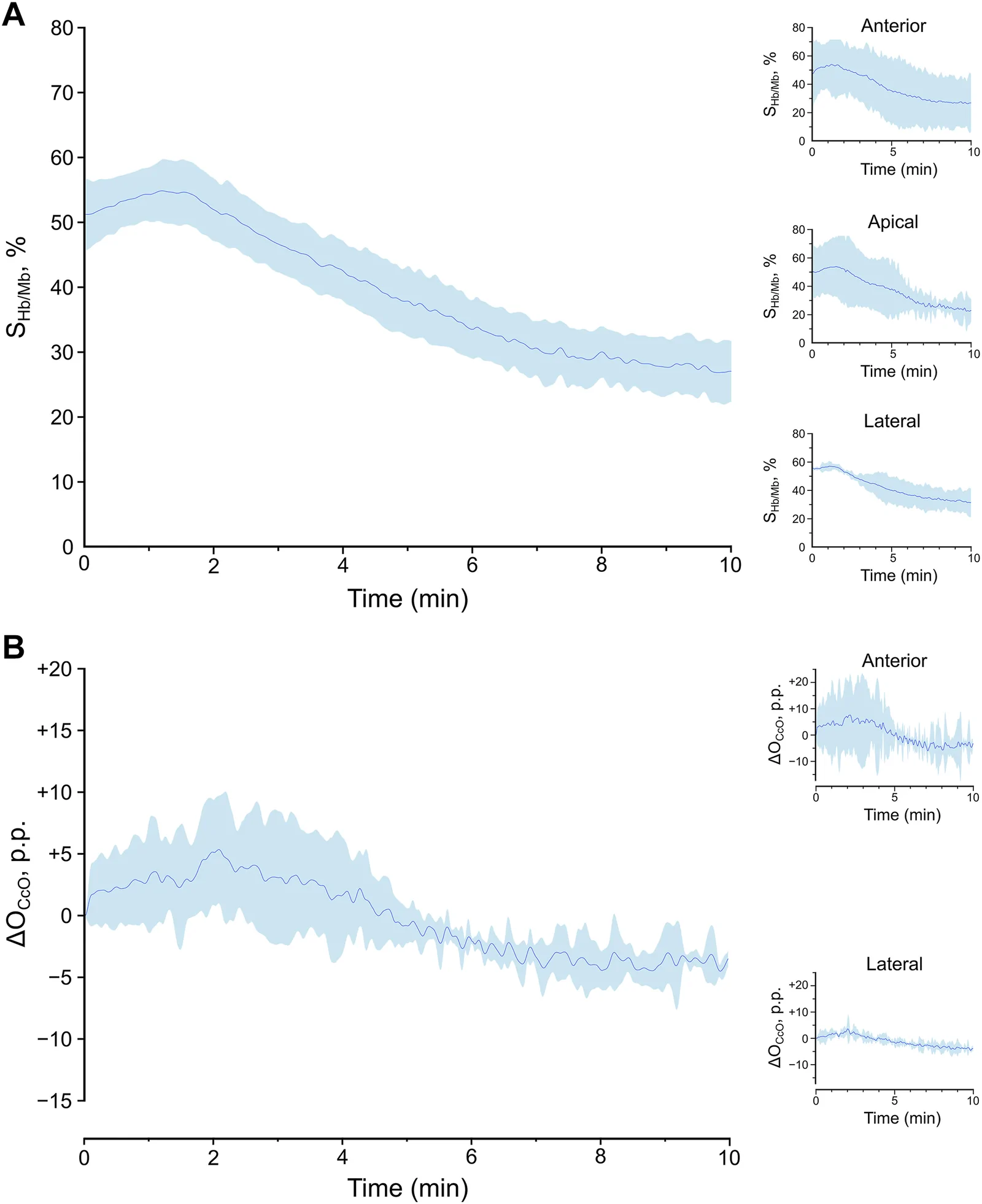
Continuous myocardial hemoglobin (Hb)/myoglobin (Mb) oxygenation and cytochrome c oxidase (CcO) oxidation ratio monitoring using the SpectroCor monitoring device. Data collected during 10 minutes after blood oxygenation and during controlled induction of reduced myocardial oxygenation in the experimental porcine cardiopulmonary bypass model in a single animal averaged over 3 repeated ventilator turn-offs. The plots show the measurement means with their 95% CIs. (A) Myocardial Hb/Mb oxygenation (S_Hb/Mb_) monitoring. Large panel: Combined data from all monitors. Small panels: individual data from all monitors and their respective sensors: Anterior, Apical, and Lateral. (B) Accuracy of CcO oxidation ratio by the SpectroCor monitoring devices and sensors. Large panel: mean percentage point (p.p.) variations in measurements of CcO oxidation changes (ΔO_CcO_) during the indicated time frame as combined from the two monitors and their respective sensors (Anterior and Lateral). Small panels: data from individual monitors and their sensors.

Figure 4A shows the combined readouts spanning the duration of 1 experiment in a single animal, from all sensors, and averaged over 3 repeated ventilator turn-offs. The SpectroCor measurement system repeatedly performed exceptionally well under a wide range of myocardial oxygenation states and independently of monitor placement. The measured combined changes in O_CcO_ during the 10-minute course of 1 experiment in a single animal, averaged over 3 repeated ventilator turn-offs, are shown in Figure 4B.

## Comment

We evaluated the SpectroCor device prototype for real-time monitoring of myocardial oxygenation and mitochondrial function using a porcine model with controlled oxygenation via CPB. Validated against measurements from arterial and venous samples with a standardized blood gas analyzer, our results demonstrated that the SpectroCor monitoring device accurately measures and tracks S_Hb/Mb_ changes during varying levels of myocardial ischemia. Although blood gas analysis reflects compartmental rather than cellular oxygenation, time-matched comparisons support the device’s accuracy.

Our results also demonstrated that the device is capable of simultaneous measurement of mitochondrial CcO activity, thus offering insight into myocardial oxygen metabolism at the cellular level. Monitoring both oxygen delivery and utilization provides a comprehensive view of myocardial metabolism, potentially helping individual tailoring of cardioprotective strategies including cardioplegia composition, timing, dosing, and temperature.

Our porcine CPB model allowed simulation of graded ischemia. Chosen for porcine similarity to human cardiac physiology, the model's results obtained here are thus highly relevant to clinical scenarios during cardiac surgery and CPB. While acceptable for a proof-of-concept, we acknowledge the small number of animals as a limitation.

In conclusion, the SpectroCor monitoring device demonstrated strong potential for improving clinical intraoperative myocardial monitoring, reducing risks of hypoxia or hyperoxia, and guiding personalized interventions. Further clinical validation is needed to confirm its broader application in cardiac and critical care settings. Following the device validation, myocardial oxygenation during aortic cross-clamping will be evaluated in future study designs.

## Freedom of Investigation

The SpectroCor monitoring devices used in this study were donated for the study. The authors retained full control of the study design, methods, outcome parameters, data collection, analysis, publication decisions, and preparation of the manuscript.

## Disclaimer

The Society of Thoracic Surgeons, The Southern Thoracic Surgical Association, and The Annals of Thoracic Surgery Short Reports neither endorse nor discourage the use of the new technology described in this article.
